# iTRAQ-based quantitative proteomic analysis reveals the distinct early embryo myofiber type characteristics involved in landrace and miniature pig

**DOI:** 10.1186/s12864-016-2464-1

**Published:** 2016-02-25

**Authors:** Xumeng Zhang, Yaosheng Chen, Jinchun Pan, Xiaohong Liu, Hu Chen, Xingyu Zhou, Zhuning Yuan, Xilong Wang, Delin Mo

**Affiliations:** State Key Laboratory of Biocontrol, Sun Yat-sen University, Guangzhou, 510006 Guangdong China; Guangdong Laboratory Animals Monitoring Institute, Guangzhou, 510663 Guangdong China

**Keywords:** Embryonic myogenesis, iTRAQ, Muscle proteome, Pig, Quantitative proteomics

## Abstract

**Background:**

Pig (Sus scrofa) is a major source of dietary proteins for human consumption and is becoming a valuable model in agricultural and biomedical research. The recently developed isobaric tag for relative and absolute quantitation (iTRAQ) method allows sensitive and accurate protein quantification. Here, we performed the first iTRAQ-based quantitative proteomic analyses of Landrace (LR) and Wuzhishan (WZS) pig longissimus dorsi muscle tissues during early embryonic development.

**Results:**

The iTRAQ-based early embryonic longissimus dorsi muscle study of LR and WZS ranging from 21 to 42 days post coitus (dpc) identified a total of 4431 proteins from17,214 peptides, which were matched with 36,4025 spectra at a false discovery rate of 5 %. In both WZS and LR, the largest amount of differentially expressed proteins (DEPs) were found between 28 and 35 dpc. 252 breed-DEPs were selected by GO analysis, including 8 myofibrillar proteins. Only MYHCI/IIA mRNA were detected due to early embryonic stages, and significantly higher expression of them were found in WZS during these 4 stages. MYHCI was first found in WZS at 28 dpc and expressed in both breeds at later stages, while MYHCII protein was not detected until 35 dpc in both breeds. Thus, 33 myogenic breed-DEPs selected from last two stages were analyzed by STRING, which showed that some myofibrillar proteins (MYH1, TPM4, MYH10, etc.) and functional proteins (CSRP2, CASQ2, OTC, etc.), together with candidate myogenic proteins (H3F3A, HDGFRP2, etc.), probably participate in the regulatory network of myofiber formation.

**Conclusion:**

Our iTRAQ-based early embryonic longissimus dorsi muscle study of LR and WZS provides new data on the in vivo muscle development distinctions during early embryonic development, which contributes to the improved understanding in the regulation mechanism of early myogenesis in agricultural animals.

**Electronic supplementary material:**

The online version of this article (doi:10.1186/s12864-016-2464-1) contains supplementary material, which is available to authorized users.

## Background

Pig is a major dietary protein source for human consumption and valuable model in agricultural and biomedical research [1–3], however, few studies concerning pig proteome were reported recently. With high-throughput technologies for protein profiling developing, great deal of molecular data will contribute to the understanding of pig early embryonic myogenesis. Pig myogenesis is clearly a biphasic phenomenon with primary myofibers forming from 35 to 55 dpc, followed by secondary myofibers forming around each primary myofiber between 50 and 90 dpc [4]. The muscle growth is predominantly determined during prenatal skeletal muscle development [5–7].

Skeletal muscle of postnatal pig consists of four major fiber types with type-specific expression of corresponding myosin heavy chain (MYHC) isoforms and specific metabolic pattern, namely slow-oxidative type I, fast-oxidative type IIa, fast oxidative-glycolytic type IIx and fast-glycolytic IIb [8, 9]. Slow oxidative type I myofibers are hypothesized to be beneficial for pork quality, while PSE (pale, soft, exudative) pork is often associated with higher percentage of MYHC IIb fibers [10–12]. Although myofiber types are influenced by neuromuscular activity, mechanical loading, and hypothyroidism, myofiber composition is of high heritability in pigs [13, 14].

High-throughput and shotgun-based proteomic techniques are increasingly utilized to improve proteome coverage and produce protein catalogues [15]. iTRAQ-mediated shotgun proteomics allows the relative quantification of peptides from eight samples simultaneously, and has proven to be a successful tool in protein biomarker discovery in biomedical field [16, 17]. Recent studies have reported 542 proteins in porcine muscle concerning gender and diet dephytinization by iTRAQ [18]. In other livestock species, the analysis of bovine skeletal muscle by iTRAQ identified changes in protein abundance between tender and tough meat from bovine longissimus thoracic muscle [19].

With the goal to find out whether early embryo proteomic distinctions exist in pig breeds differing in meat quality and gain further insights into the mechanism. 21, 28, 35, 42 dpc embryos/fetuses of two pig breeds were employed: LR, an improved large pig breed, is characterized by high lean meat percentage, fast growing muscle and high body weight [7, 20, 21] and WZS pig, originates from Hainan Island, China, is known for its superior meat quality, and is considered useful for medical research due to its small size (adult weights < 25 kg) [22]. The longissimus dorsi muscle proteome was analyzed by iTRAQ. Myofiber type characteristics were studied by real-time quantitative PCR and western blot (WB). Through these assays, we wanted to uncover the early embryo proteomic distinctions in two pig breeds and elucidate the mechanism underlying muscle development, which may contribute to the understanding of early embryonic myofiber formation in agricultural animals.

## Results

### DEPs of adjacent stages in WZS and LR

**Fig. 1:**
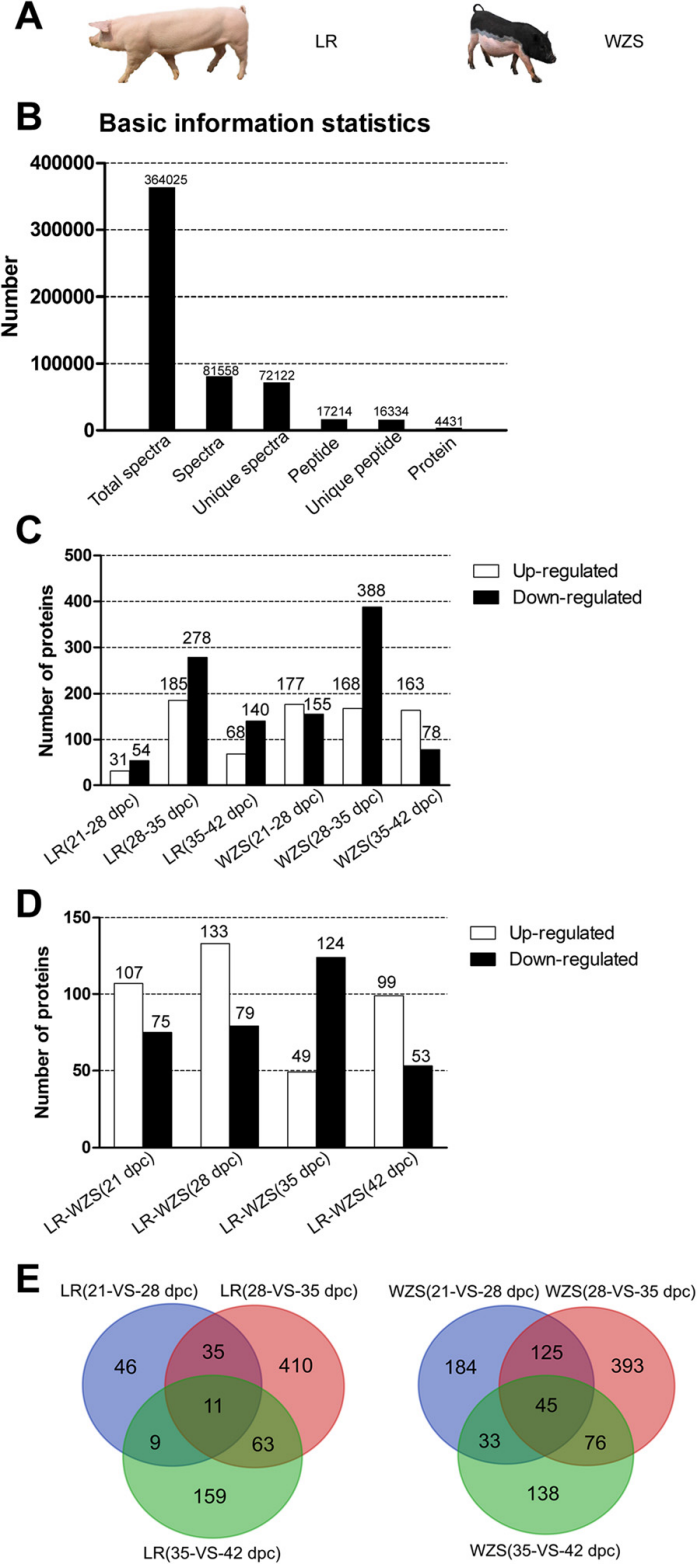
Basic information of iTRAQ. **a** The photo of two pig breeds used in this study. LR is much larger than WZS. **b** Basic information of protein identification. **c** Up or down-regulated proteins within WZS and LR at adjacent stages or **d** between at same stages. **e** Venn diagrams showing the number of total DEPs identified by MS/MS (≥95 % confidence) within WZS or LR at adjacent stages

In total, 4431 proteins were identified from 17,214 peptides, which were matched with 36,4025 spectra at a false discovery rate of 5 % (Fig. 1b, Additional file 1: Figure S1.).

The largest amount of up or down-regulated proteins within WZS (168 proteins up and 388 proteins down-regulated) and LR (185 proteins up and 278 proteins down-regulated) were found in the comparison between 28 and 35 dpc (Fig. 1c), and morphologic observations also showed that myofibers didn’t appear until 35 dpc in both breeds (Additional file 2: Figure S2). Taken together, we deduced that 28–35 dpc is important for embryonic muscle development for pig breeds, during which a number of proteins participated in and the myofibers began to form. In the comparisons between LR and WZS at same stages, the largest number of up-regulated DEPs (133 up-regulated DEPs) were found at 28 dpc and the largest number of down-regulated DEPs (124 down-regulated DEPs) were at 35 dpc (Fig. 1d), which indicated that early development of WZS and LR embryos were distinctly regulated by more DEPs during this period than the other two stages. Thus we speculated that 28–35 dpc is also important for embryonic development between breeds. Significantly more DEPs were found in WZS at adjacent stages studied (Fig. 1d, e). Over 9 myogenic terms (muscle cell differentiation, skeletal muscle myosin thick filament assembly, etc.) for biological process (BP) were found significantly enriched in WZS and only two (muscle system process and muscle contraction) were found significantly enriched in LR at adjacent stages (Additional file 3: Table S1), suggesting that distinct skeletal muscle development pattern has arisen at early embryonic stages between WZS and LR.

### DEPs between WZS and LR at same stages

**Fig. 2:**
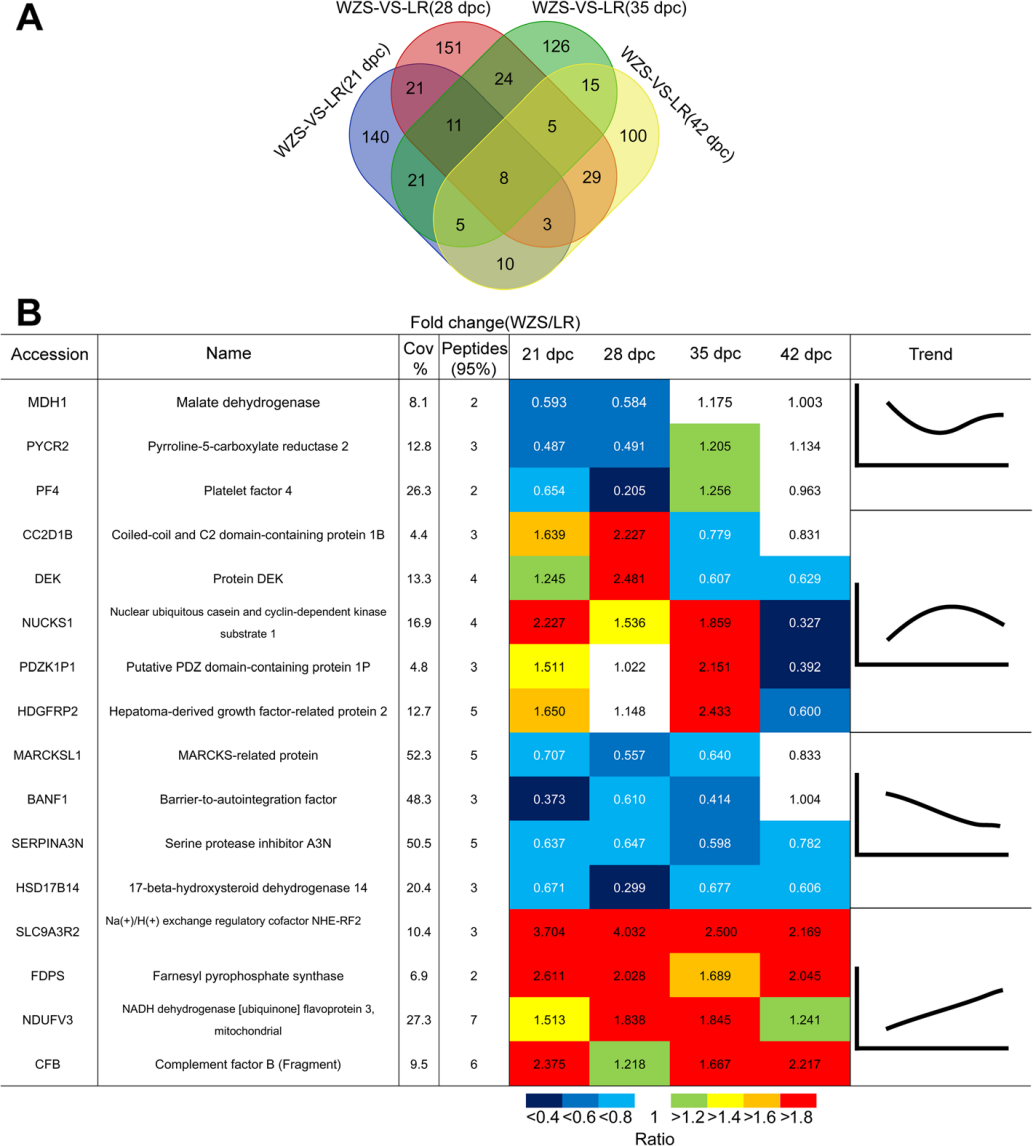
DEPs between WZS and LR at 4 stages. **a** Venn diagram showing the number of total DEPs identified by MS/MS (≥95 % confidence) between WZS and LR at 21,28,35,42 dpc. **b** Significant DEPs Identified in WZS and LR. Different color represents different fold change

Protein quantification based on the relative amounts of the different iTRAQ labels was obtained for all the identified proteins with two or more unique peptides. The datasets for differences in protein levels between WZS and LR at same stages were analyzed then. The largest amount of DEPs were found in 28 dpc (252 DEPs) (Fig. 2a, Additional file 4: Figure S3, Additional file 5: Table S2). While at 21 and 35 dpc, smaller but still great amount of DEPs were present (219 and 215 DEPs, respectively). The smallest amount of DEPs were found at 42 dpc (175 DEPs).

After analyzing common DEPs between WZS and LR at same stages, 16 significant DEPs were selected with 4 trends based on ratio of DEPs’ expression level (WZS/LR) and GO terms for BP were also analyzed in each trend (Fig. 2b, Additional file 6: Figure S4, Additional file 3: Table S1, Additional file 5: Table S2): 1) proteins down-regulated at 21–28 dpc and up-regulated at 35–42 dpc (MDH1, PYCR2, PF4); involved GO terms for BP: energy production/conversion, amino acid transport and metabolism, 2) proteins up-regulated at 21–35 dpc and down regulated at 35–42 dpc (CC2D1B, DEK, HDGFRP2, etc.); involved GO terms for BP: signal transduction and DNA-dependent transcription, 3) proteins down-regulated at 21–42 dpc (MARCKSL1, BANF1, HSD17B14, etc.); involved GO terms for BP: positive regulation of cell proliferation and DNA integration, 4) proteins up-regulated at 21–42 dpc (FDPS, NDUFV3, CFB, etc.); involved GO terms for BP: coenzyme transport and metabolism and metabolic process (Additional file 3: Table S1).

### Myogenic DEPs between WZS and LR at same stages

**Fig. 3:**
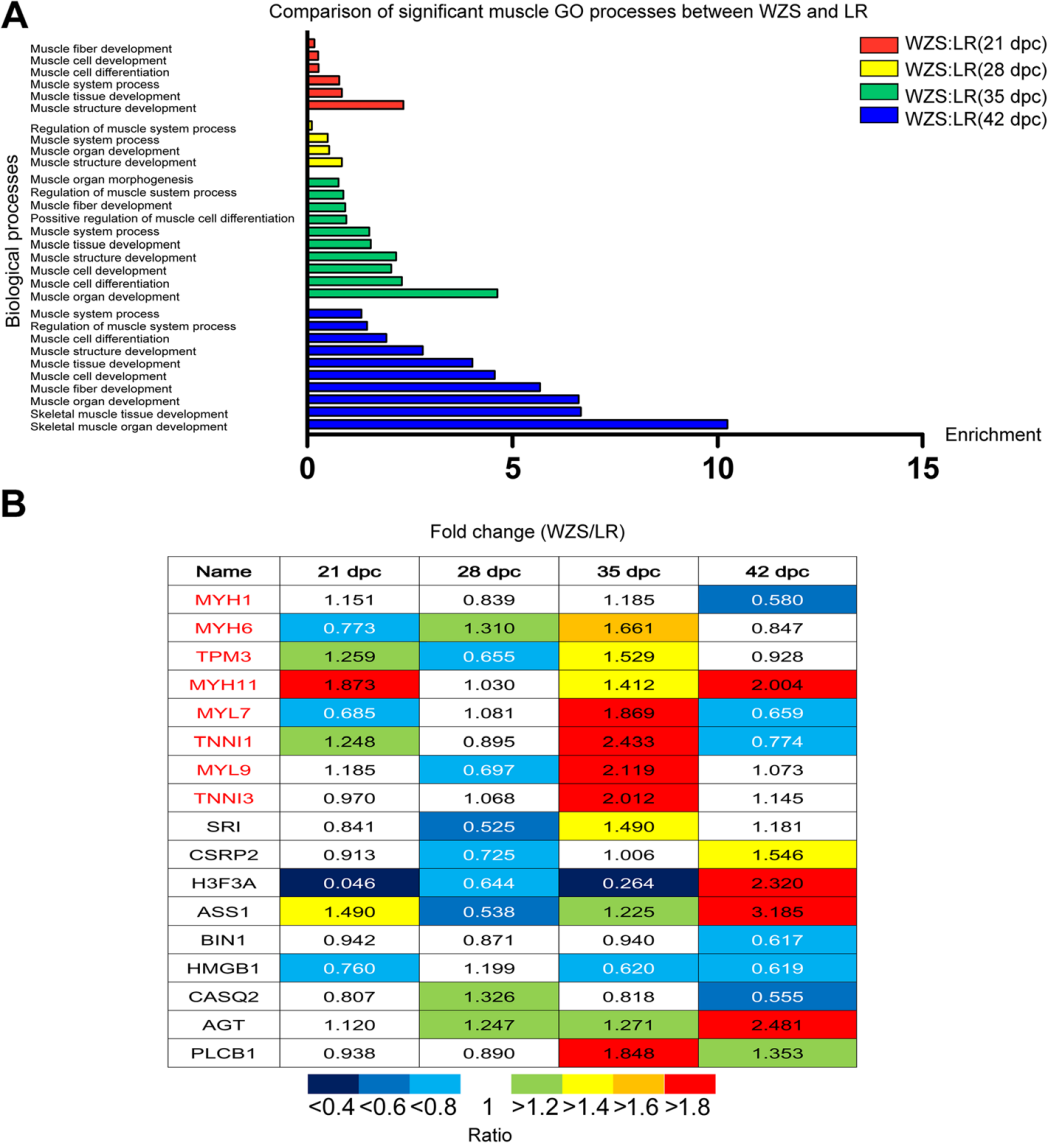
Comparison of significant myogenic GO and DEPs between LR and WZS. **a** Comparison of significant muscle GO biological processes between WZS and LR. We chose the lowest 4–10 *p*-value of myogenic GO terms and converted them to the value of enrichment by calculating the absolute value of log_2_ at each stage. **b** Comparison of significant muscle DEPs between LR and WZS. Different color represents different fold change. Myofibrillar proteins are marked red

In term of COG database, the function classification of identified protein sequences were listed (Additional file 7: Figure S5, Additional file 8: Table S3, Additional file 9: Table S4). Top myogenic GO terms for BP with the minimal *p* values were chosen between WZS and LR at same stages, such as muscle system process, muscle cell differentiation and muscle fiber development, etc. The highest enrichment of myogenic BP were found at 42 dpc (Fig. 3a), six of which with *p* value less than 0.05 (Additional file 10: Table S5). While at 35 dpc, lower enrichment of myogenic BP was present and only one term with *p* value less than 0.05. At 21 and 28 dpc, no terms with *p* value less than 0.05 were found.

From the proteins involved in the top 5 myogenic GO terms for BP, 17 myogenic DEPs between WZS and LR were chosen (1.5-fold change) (Additional file 11: Table S6). Taken together, most myogenic DEPs (WZS/LR) were down-regulated at 21 or 28 dpc and up-regulated at 35 dpc, among which 8 myofibrillar proteins showed distinct expression pattern (Fig. 3b). Only 3–5 myogenic DEPs were found at 21 and 28 dpc, while at stage 35 and 42 dpc, 9–12 myogenic DEPs were present (Additional file 11: Table S6, Additional file 12: Table S7).

### Myofiber type distinction at early embryonic stages

There are 8 myofibrillar proteins showing distinct expression between WZS and LR. In order to validate whether myofiber type distinction existed, 4 types of MYHC isoforms were examined by qPCR and only MYHCI/IIA were detected in WZS and LR. Both of them were found with significantly higher expression in WZS covering 4 stages.

**Fig. 4:**
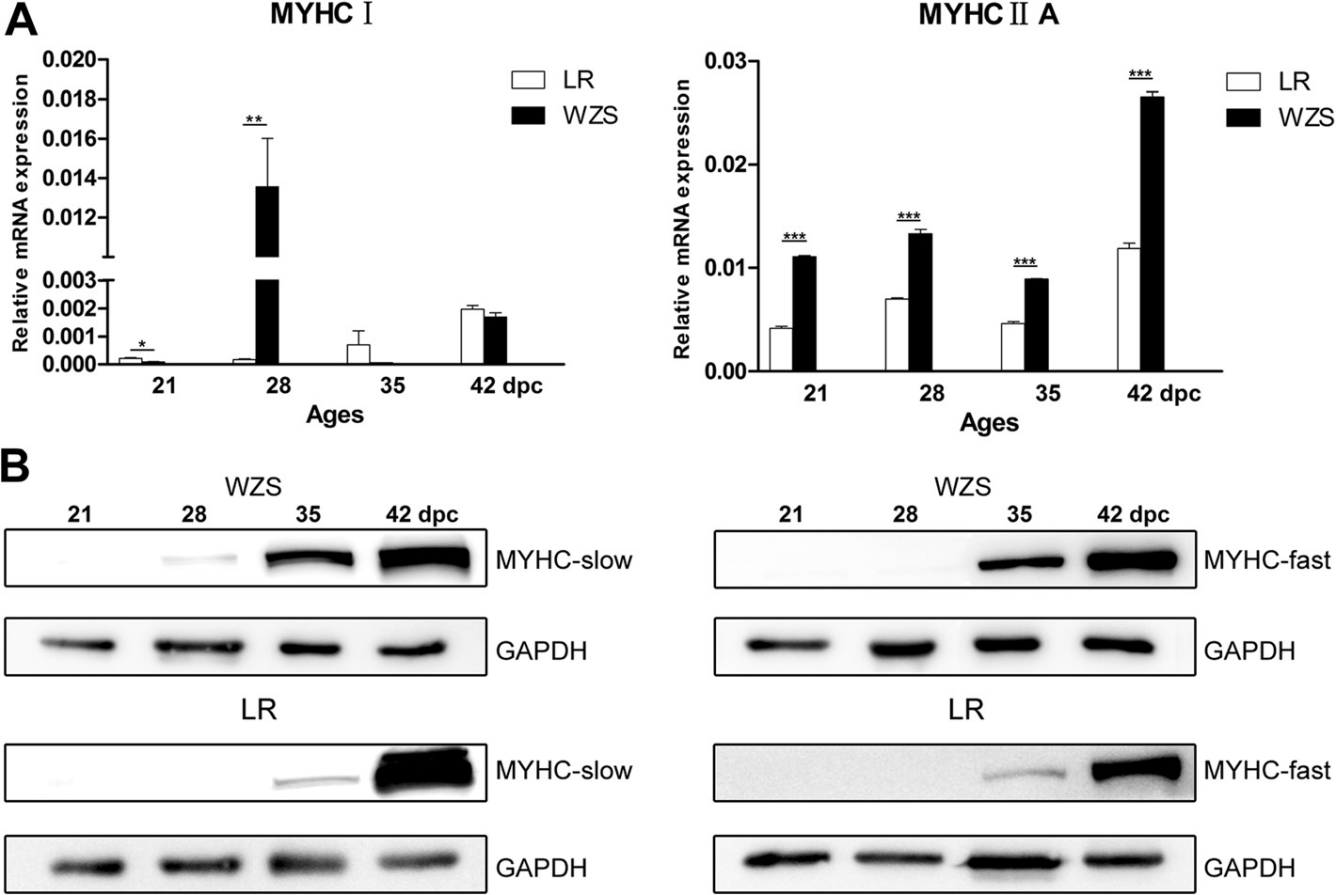
Validation of distinct embryonic myofiber type development between WZS and LR. **a** qPCR of MYHCI and MYHCIIA in WZS and LR at 4 stages.* *p* < 0.05, ** *p* < 0.005, ***p < 0.001 (LR vesus WZS, *n* = 3). **b** WB of LR and WZS at 28, 35, 42 dpc. GAPDH was used as control. Type II and typeI MYHC protein was detected by anti-myosin (skeletal, fast or slow) antibodies

To further validate the distinction of myofiber development, MYHCI/II proteins were detected by WB. The expression of MYHCII protein was not detected until 35 dpc in both breeds, while MYHCI was first found in WZS at 28 dpc and expressed in both breeds at later stages. It’s interesting that both MYHCI and MYHCII proteins were significantly higher expressed in WZS at 35 dpc, while until 42 dpc, MYHCI was higher expressed in LR (Fig. 4b). Morphologic observations showed that myofibers didn’t appear until 35 dpc in both breeds and myofibers in WZS at 35–42 dpc tended to be larger in size and more in number than those in LR (Additional file 2: Figure S2).

Taken together, at early embryonic stages, myofiber type distinction has been existed between two breeds and WZS showed stronger trend of myofiber differentiation during 35–42 dpc.

### Protein interaction networks of significantly DEPs

**Fig. 5:**
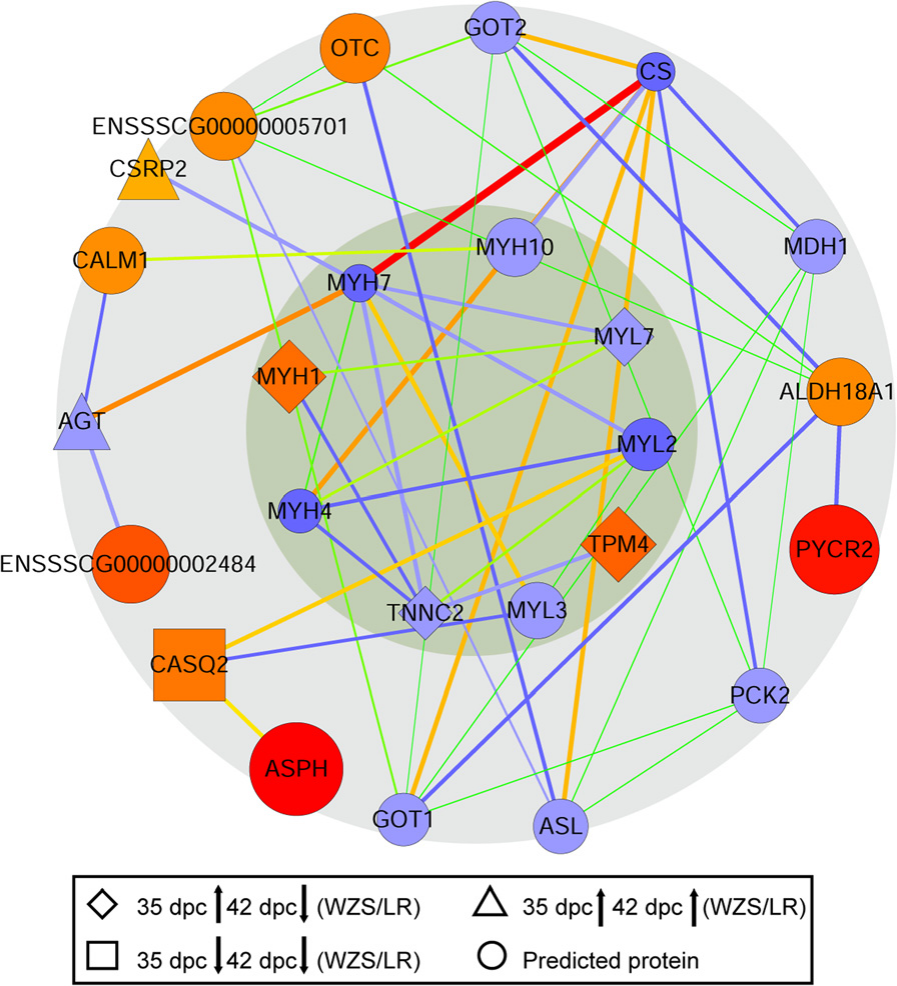
Interaction network of important myogenic proteins identified by iTRAQ. Protein interactions were predicted in the website of STRING and the interaction network were illustrated by Cytoscape software. There are two cycles in the figure. Myofibrillar proteins are in the inner cycle; other proteins predicted to have interactions with the inner cycle proteins are listed in the outer cycle. Thicker line represents stronger interaction and bigger shape size represents that the protein may interact with more proteins. The strongest to lowest interaction level is marked by red to blue in shapes and lines. Different forms of shape represent different expression trends in 35 and 42 dpc, which are listed in the figure

Protein interactions were predicted in the website of STRING and the interaction network was illustrated by Cytoscape software. There are two cycles in the figure (Fig. 5). Myofibrillar proteins are in the inner cycle; other proteins that were predicted to have interactions with inner cycle proteins are in the outer cycle. As predicted, several strong interactions were found between outer (Ornithine Carbamoyltransferase, Angiotensinogen, Sorcin, Calsequestrin 2) and inner cycle myofibrillar proteins (Troponin C, Myosin regulatory light chain 7, Myosin 4, Myosin 1, Tropomyosin alpha-4) (Fig. 5). Since most of the outer cycle proteins were up or down regulated at both 35 and 42 dpc, and most of the inner cycle proteins were up-regulated at 35 dpc and down-regulated at 42 dpc, it can be reasonably speculated that the outer cycle proteins regulate the expression profile of inner cycle proteins and the distinct expression of outer cycle proteins in WZS and LR may result in distinct characteristics of myofiber formation.

## Discussion

In this study, a comprehensive evaluation of proteomic profile in 21, 28, 35, and 42 dpc of LR and WZS pig were performed, providing new data about distinctions in vivo during early embryonic muscle development. The results showed higher and earlier expression of type I/II MYHC proteins in WZS, which indicate distinct regulation network of early embryonic myofiber development between pig breeds differing in meat quality.

### Distinct muscle development pattern in WZS and LR

In this study, porcine embryonic longissimus dorsi muscle proteomic analysis covered the process of primary myofiber formation. In comparison of WZS and LR at adjacent stages, much more DEPs and significantly enriched myogenesis BP terms were found in WZS. In comparison between WZS and LR at same stages, although large amount of DEPs were found in 21 and 28 dpc, no significant myogenic GO terms were found. While at 35 and 42 dpc, though the number of DEPs dropped significantly, higher enrichment of myogenic GO terms were found. These findings indicated that 21–28 dpc may be important stages for the preparation of early muscle development and the DEPs involved in these stages probably participate in the distinct muscle development process between WZS and LR since 35 dpc.

### Distinct myofiber characteristics between WZS and LR

To further investigate the myogenic DEPs between WZS and LR, we analyzed the proteins involved in top myogenic GO terms for BP. Many myofibrillar proteins (myosin, troponin, etc.) are known to exist as multiple isoforms [23]. It has been demonstrated that some myofibrillar isoforms are differentially expressed in various muscles and fiber types. Myosin makes up about one-third of the total muscle proteins and is known to produce multiple isoforms. Different myosins involved in differentiate progress of distinct muscle fiber types: type I (slow-twitch, red muscle, oxidative) (MYH7), type IIa (fast-twitch, red muscle, oxidative) (MYH2), and type IIb/IIx (fast-twitch, white muscle, glycolytic) (MYH1) [8, 24].

In this study, multiple differentially expressed myosin proteins were found, among which MYH11, MYL9, MYH6, TNNI1, MYL7, TPM3, TNNI3 were up-regulated in WZS at least 1 stage we studied. In addition, mRNA and protein level of MYHCI/II isoforms also showed higher expression in WZS at all 4 stages. Longissimus dorsi muscle is a fast-twitch glycolytic muscle involved in voluntary movements of the back. It was reported that faster growing commercial pig breeds with more muscle contain high proportions of type IIb/IIx glycolytic fibers (75–86 %) [21]. No studies concerning WZS myofiber composition were reported, while previous studies indicated that skeletal muscles of Meishan pig, another Chinese indigenous pig breed, weighed only 36.1 kg in average before mating [25], comprises of higher proportion of slow fibers at slaughter, which was hypothesized to be the major attribute to the superior meat quality [26]. As we mentioned before, myofiber composition is of high heritability in pigs. Previous study concerning later embryonic stages to adult pig shows that myofiber types at different stages and different part of the longissimus dorsi muscle have distinct composition of myofiber types [27], while this study focused on the early embryo stages including primary myofiber formation. Thus, we speculated that the myofiber type distinction began to show up between LR and WZS in early embryonic stages and changing through later developmental stages, which determined the distinct adult myofiber composition between LR and WZS.

### DEPs involved in the regulatory network of early muscle development

Several DEPs showed significant changes during these stages. CSRP2 associates with meat quality traits with CSRP1 and CSRP3 in pigs [28]. This protein was down-regulated at 28 dpc and up-regulated at 42 dpc in WZS. H3F3A (Histone H3.3) dropped 74-96 % from 28–35 dpc but increased 2.5 folds at 42 dpc in WZS. Hepatoma-derived growth factor-related protein 2 (HDGFRP2) was up-regulated 2.4 fold at 35 dpc and dropped 60 % at 42 dpc in WZS, and it includes an N-terminal PWWP domain that binds to methyl-lysine-containing histones, with specific binding of this protein to tri-methylated lysines 36 and 79 of histone H3, and di- and tri-methylated lysine 20 of histone H4 [29]. It is reported that myoblast determination protein (MyoD) determines cell fate and facilitates differentiation-dependent gene expression through chromodomain helicase DNA binding protein 2 (CHD2) -dependent deposition of H3.3 at myogenic loci prior to differentiation [30]. Recent study found that selective H3.3 incorporation is essential for establishing specific modifications in myogenic genes, suggesting that the incorporation of specific histone H3 variants determines the lineage potential of myogenesis [31]. Thus the distinct expression of H3F3A and HDGFRP were very likely to regulate the distinct early muscle development between WZS and LR.

Several myofibrillar proteins and functional proteins involving in strong interaction networks were found by STRING analysis (Fig. 5). For example, SRI plays a role in the translation of mechanical cues applied to myofibers into paracrine signals, which in turn modulate satellite cell functions and support muscle growth [32]. It was down-regulated at 28 dpc and up-regulated at 35 dpc in WZS. CASQ2, a calcium binding protein that stores calcium for muscle function [33], was up-regulated since 21 dpc in WZS. The gene of OTC was mapped closely to Duchenne muscular dystrophy, which may play a role in myogenesis [34]. Taken together, most myogenic DEPs were down-regulated at 21 or 28 dpc and up-regulated at 35 or 42 dpc in WZS, therefore, it’s reasonable to believe that these functional proteins participated in the regulatory network of distinct myofiber development process. In adult mice, a selective accumulation of MyoD transcript in the fast-twitch muscles and myogenin transcripts in the slow-twitch skeletal muscles suggests that fibre characteristics may be affected by the ratio of the different muscle regulatory factors [35, 36], but few studies about regulation mechanism of myofiber formation in agricultural animals are reported.

Based on the above findings, further studies could focus on when and how the myogenic related factors differentially express between the two pig breeds in embryos. As in our study, the expression of H3F3A in LR was significantly higher than that in WZS (about 20 folds) at 21 dpc. Thus we speculate that this histone modification may play a key role in determining muscle development in embryos. As recent study shows that mice embryonic early skeletal muscle lineage potential is established through a selective incorporation of specific H3 variants that governs the balance of histone modifications [31].

## Conclusion

In summary, this iTRAQ-based early embryonic muscle study of WZS and LR showed that the distinction of myofiber characteristics began in the early embryonic stages, which probably result in distinct myofiber type composition after birth. Our study contributes to the understanding of early embryonic myofiber formation and differentiation.

## Methods

### Ethics statement

All experiments were carried out according to China Council on Animal Care and the protocols used were approved by the Animal Care and Use Committee of Guangdong Province, China. Approval ID or permit numbers are SCXK (Guangdong) 2011–0029 and SYXK (Guangdong) 2011–0112 (Additional file 13).

### Animals

Twenty-four purebred sows with the same genetic background of LR and WZS were artificially inseminated with semen from the same purebred boars. For each breed in prenatal stages, three sows per time point were slaughtered at 21, 28, 35 and 42 dpc after insemination, and embryos/fetuses were collected as described before [37]. Whole embryos of 21 dpc (Additional file 14: Figure S6) and longissimus dorsi muscle tissues from fetuses of left stages were used. These samples were snap-frozen in liquid nitrogen and stored until further use.

### Protein isolation and iTRAQ labeling

For protein analyzing, tissues were processed as described before [38]. As the amount of protein in one embryo was small (especially at 18 & 21 dpc), we used a single pooling strategy in our study as previous similar studies reported [39–41]. Previous study also pointed that the pooling strategy is appropriate in experiments with low sample yield, and could reduce the biological variance, increasing the power to detect changes [42]. Three embryos/fetuses of different sows were mixed as a pool for each breeds at every stages. 100 μg protein for each sample was digested with Trypsin Gold (Promega, USA) at a protein: trypsin ratio of 20:1 at 37 °C for 4 h. Then peptides were dried via vacuum centrifugation. The samples were labeled with 113 (LR 21 dpc), 114 (LR 28 dpc), 115 (LR 35 dpc), 116 (LR 42 dpc), 117 (WZS 21 dpc), 118 (WZS 28 dpc), 119 (WZS 35 dpc) and 121 (WZS 42 dpc) iTRAQ reagent, respectively, by incubation at room temperature for 2 h. To reduce sample complexity, strong cation exchange (SCX) chromatography, using an off-line SCX cartridge and buffers supplied by ABI, was carried out for cell lysate-derived peptides.

### LC-ESI-MS/MS analysis by LT Q-Orbitrap HCD

The peptides were subjected to nanoelectrosprayionization followed by tandem mass spectrometry (MS/MS) in a LTQ Orbitrap Velos (Thermo Fisher, USA) coupled online to the HPLC (Shimadzu, Kyoto, Japan) and processed as described before [43]. For MS scans, the m/z scan range was 350 to 2000 Da. The experiment was repeated three times, and the results were categorized as 113–121 groups.

### Database search, protein identification and quantification

The MS/MS data were searched against the Ensembl: Sus_scrofa.Sscrofa1 0.2.69.pep.all.fa (25152 sequences) database for peptide identification and quantification using the Mascot 2.3.02.

For protein identification, a mass tolerance of 6 Da (ppm) was permitted for intact peptide masses and 0.5 Da for fragmented ions, with allowance for one missed cleavages in the trypsin digests. Gln- > pyro-Glu (N-term Q), Oxidation (M), Deamidated (NQ) as the potential variable modifications, and Carbamidomethyl (C), iTRAQ8plex (N-term), iTRAQ8plex (K) as fixed modifications. The charge states of peptides were set to 2+, 3+, 4+. Specifically, an automatic decoy database search was performed in Mascot by choosing the decoy checkbox in which a random sequence of database is generated and tested for raw spectra as well as the real database. To reduce the probability of false peptide identification, only peptides at the 95 % confidence interval by a Mascot probability analysis greater than “identity” were counted as identified. After this filter, the FDR was very low, less than 1.3 %. And each confident protein identification involve at least one unique peptide.

For protein quantitation, it was required that a protein contains at least two unique spectra. The quantitative protein ratios were weighted and normalized by the median ratio in Mascot. We set a 1.5-fold change as the threshold and *p*-value (*t*-test) < 0.05 to identify significant changes.

### RNA Extraction, and real-time quantitative PCR

To validate the iTRAQ data that myofibrillar proteins distincted from two breeds, we use real-time quantitative PCR to detect the mRNA level of type I/II MYHC isoforms. RNA was extracted from the same samples of iTRAQ analysis using NucleoSpin RNA II Extract kit (Macherey-Nagel, USA), and the quality assessed with a NanoDrop ND-1000 (Thermo Scientific, USA). cDNA was synthesized using the Transcriptor First Strand cDNA Synthesis Kit (Roche, USA).

Real-time quantitative PCR reactions were carried out in triplicate using the LightCycler 480 System (Roche, USA). The expression of each gene was normalized to that of GAPDH transcripts. Results are given as mean ± SD. The single (*), double (**) and triple (***) asterisks represent the *p* values, *p* < 0.05, *p* < 0.005 and *p* < 0.001, respectively for Student’s unpaired t tests. The following oligonucleotides were used:

MYHCI, (forward) CACTTGCTAAGAGGGACCTCTGAGTTCA; (reverse) ATCCAGGCTGCGTAACGCTCTTTGAGGTTGTAMYHCIIA, (forward) AGCCTCTTTCTTCTCCCAGGGACATTC; (reverse) ATCCAGGCTGCGTAACGCTCTTTGAGGTTGTAGAPDH, (forward) TACATGGTCTACATGTTCCAGTATG; (reverse) CAGGAGGCATTGCTGACAATCTTG

### Western blot

For analyzing type I/II MYHC protein isoforms, proteins were extracted and run on a 12 % SDS-PAGE gel and transferred onto a PVDF membrane. Anti-fast skeletal myosin antibody (Abcam, China) and anti-Slow Skeletal Myosin Heavy chain antibody (Abcam, China) were used for MYHC and anti-GAPDH-71.1 antibody (Sigma, USA) for GAPDH. The anti-mouse and anti-rabbit secondary antibody (CST, USA) was used for each experiment. Blots were visualized using a commercial enhanced chemiluminescene (ECL, USA) detection Kit (Thermo Scientific, USA).

### Bioinformatics and statistical analysis

The cellular component (CC), molecular function (MF) and biological process (BP) of the proteins identified by iTRAQ were annotated by the Blast2GO software (https://www.blast2go.com/); GO functional classification and enrichment analysis were also performed to identify GO terms that were significantly enriched in DEPs using DAVID analysis (http://david.abcc.ncifcrf.gov/). The myogenic DEPs and predicted protein interaction network was illustrated by STRING (http://string-db.org/) and Cytoscape 3.1.0 (http://www.cytoscape.org/). Relative expression of proteins was based on the ratio of the reporter ions of peptides using Mascot software. *T*-test was used to analyze the *p*-value. Moreover, the protein with the 1.5-fold change as well as the *p* value less than 0.05 was designated the DEPs. Statistical analyses were conducted with SPSS 10.0.

### Availability of supporting data

The datasets supporting the results of this article are included within the article (and its additional file(s)). The mass spectrometry proteomics data have been deposited to the ProteomeXchange Consortium via the PRIDE partner repository with the dataset identifier PXD002481.

## Additional files

Additional file 1: Figure S1. Basic information of iTRAQ. (A) Basic information statistics; (B) Distribution of protein’s sequences coverage; (C) Peptide length distribution; (D) Peptide number distribution; (E) Protein mass distribution; (F) Length of peptide (in amino acids); (G) Spectrogram matching mass error distribution. (TIF 501 kb) Additional file 2: Figure S2. (A) Hematoxylin-eosin staining and (B) immunohistochemical staining of LR and WZS in different stages. MYHC-fast (Abcam, ab7784) antibody was used in (B) which was marked by red. Scale bar = 50 μm. (TIF 7013 kb) Additional file 3: Table S1. Complete list of up and down-regulated proteins identified by iTRAQ. (XLS 1326 kb) Additional file 4: Figure S3. Protein ratio distribution between LR and WZS at 4 stages. (TIF 143 kb) Additional file 5: Table S2. List of significantly changed DEPs identified by iTRAQ between LR and WZS at 4 stages. (XLSX 20 kb) Additional file 6: Figure S4. GO categories of identified proteins. (TIF 1081 kb) Additional file 7: Figure S5. COG function classification of identified proteins. (TIF 416 kb) Additional file 8: Table S3. Detail of GO, GO2proteins and protein2GO identified by iTRAQ. (XLS 3474 kb) Additional file 9: Table S4. Terms from the muscle process ontology with *p*-value as good or better than 1 between LR and WZS at 4 stages. (XLSX 11 kb) Additional file 10: Table S5. Detail of COG identified by iTRAQ. (XLS 7958 kb) Additional file 11: Table S6. Complete list of significantly changed myogenic DEPs identified by iTRAQ between LR and WZS at 4 stages. (XLSX 45 kb) Additional file 12: Table S7. Detail of some important myogenic proteins’ interaction network identified by STRING (http://string-db.org/). (XLSX 14 kb) Additional file 13:
**ARRIVE checklist.** (PDF 1104 kb) Additional file 14: Figure S6. Embryo picture of 21 dpc (LR&WZS). (TIF 164 kb)

## Abbreviations

BP: Biological process
CC: Cellular component
CSRP2: Cysteine and glycine-rich protein 2
DEPs: Differentially expressed proteins
dpc: Days post coitus
H3F3A: Histone H3.3
HMGB1: High mobility group protein B1
LR: Landrace
LT: Lantang
MF: Molecular function
MYH1: Myosin 1
OTC: Ornithine carbamoyltransferase
SCX: Strong cation exchange
WZS: Wuzhishan
