# Supplementary figures and images for: iTRAQ-based quantitative proteomic analysis reveals the distinct early embryo myofiber type characteristics involved in landrace and miniature pig
Source: BMC Genomics. 2016 Feb 25;17:137. doi: 10.1186/s12864-016-2464-1 (PMC4766617; doi:10.1186/s12864-016-2464-1)

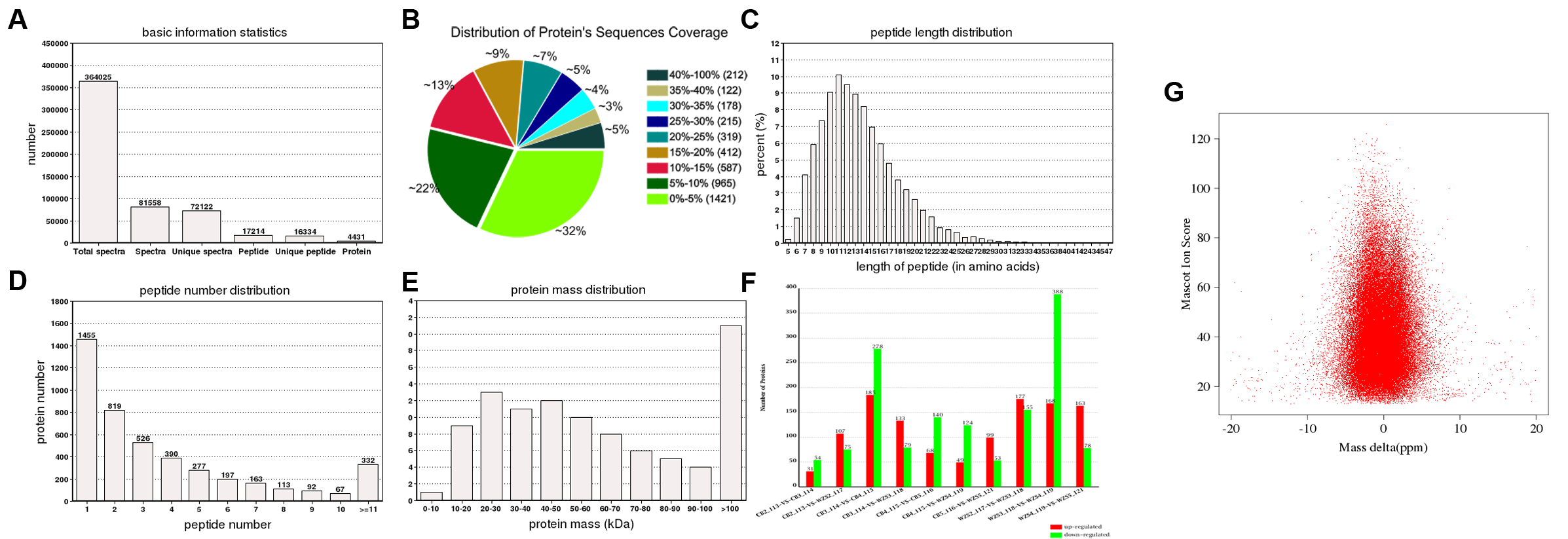

Supplement: Additional file 1: Figure S1. — Basic information of iTRAQ. (A) Basic information statistics; (B) Distribution of protein’s sequences coverage; (C) Peptide length distribution; (D) Peptide number distribution; (E) Protein mass distribution; (F) Length of peptide (in amino acids); (G) Spectrogram matching mass error distribution. (TIF 501 kb) [file 12864_2016_2464_MOESM1_ESM.tif]

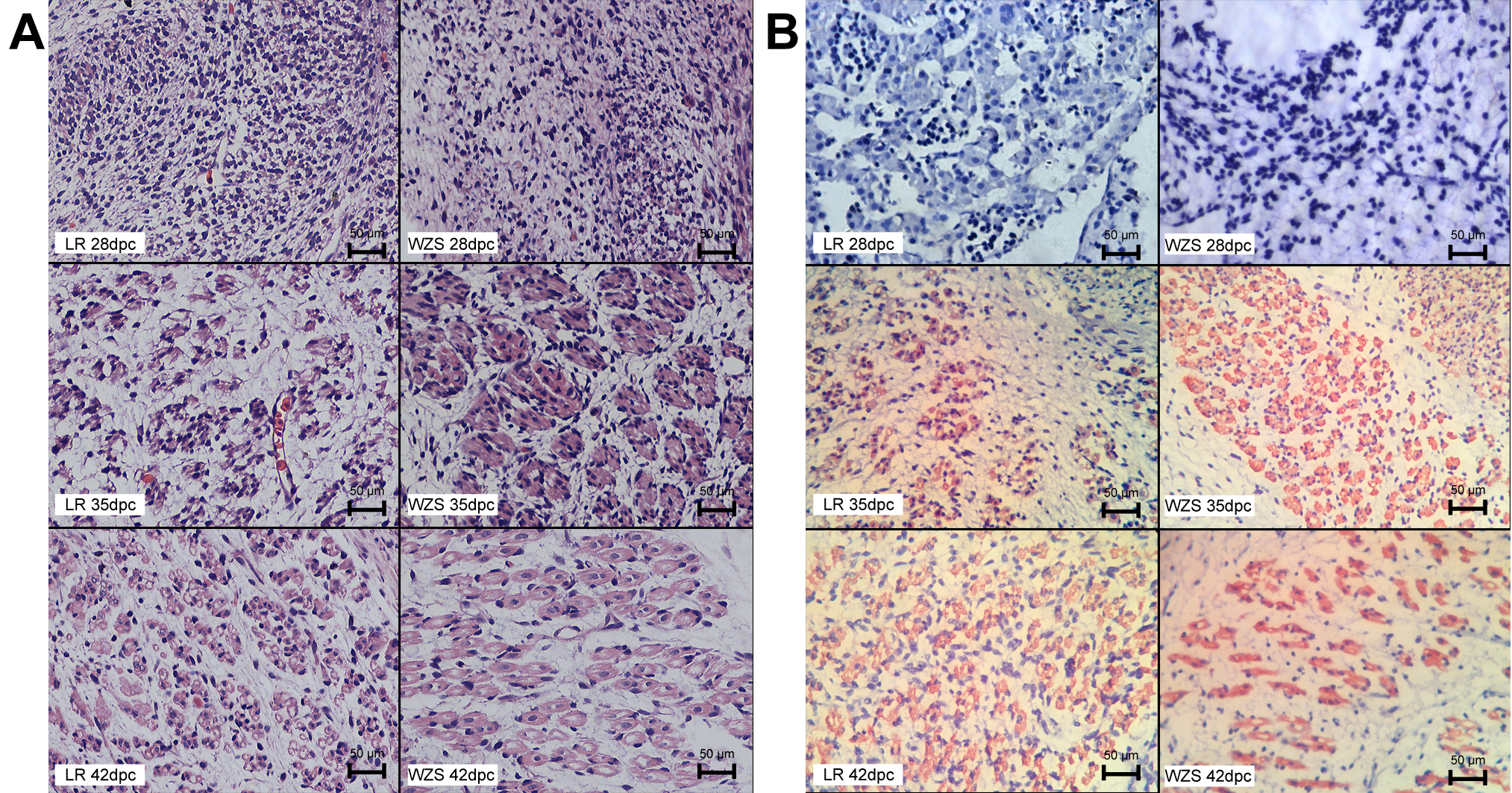

Supplement: Additional file 2: Figure S2. — (A) Hematoxylin-eosin staining and (B) immunohistochemical staining of LR and WZS in different stages. MYHC-fast (Abcam, ab7784) antibody was used in (B) which was marked by red. Scale bar = 50 μm. (TIF 7013 kb) [file 12864_2016_2464_MOESM2_ESM.tif]

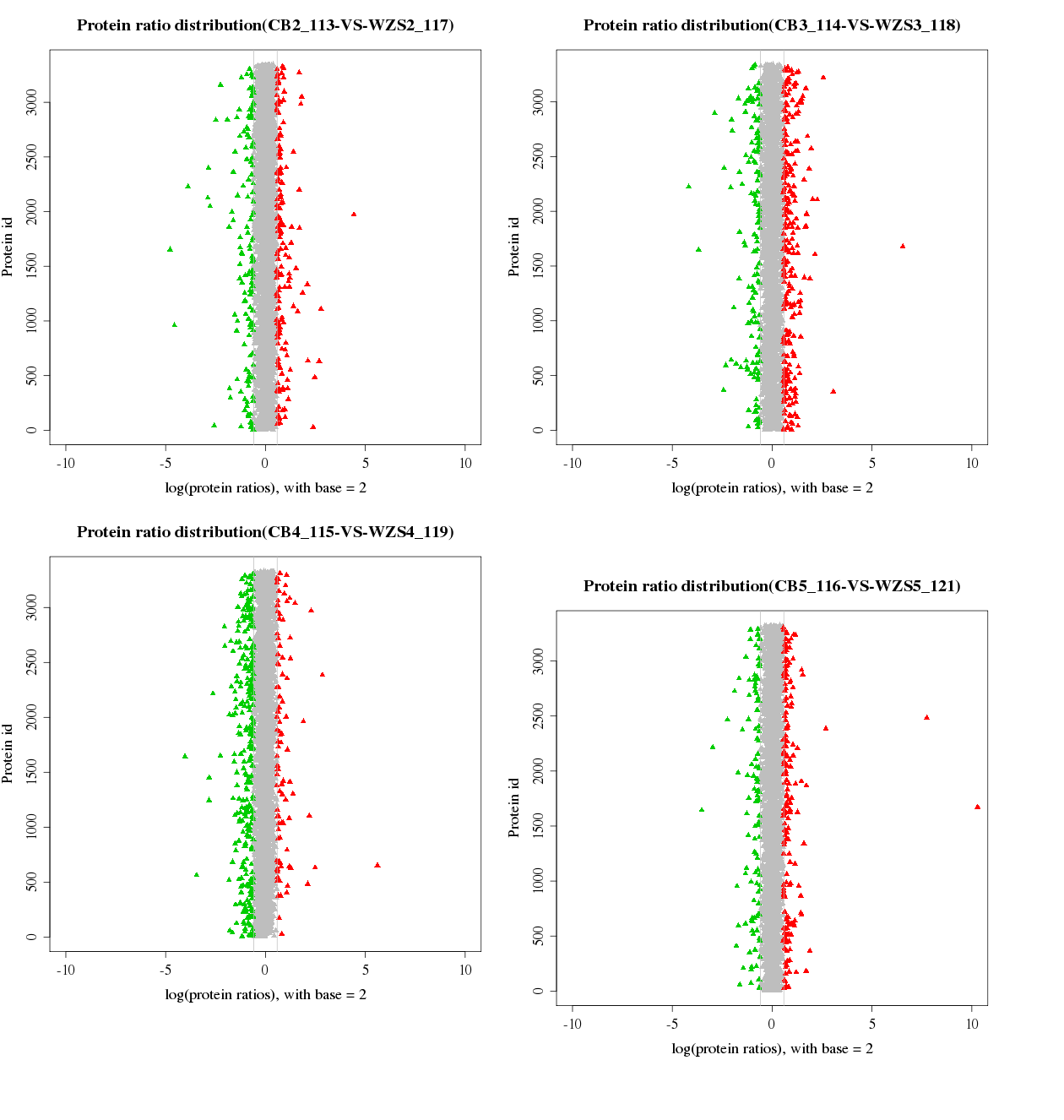

Supplement: Additional file 4: Figure S3. — Protein ratio distribution between LR and WZS at 4 stages. (TIF 143 kb) [file 12864_2016_2464_MOESM4_ESM.tif]

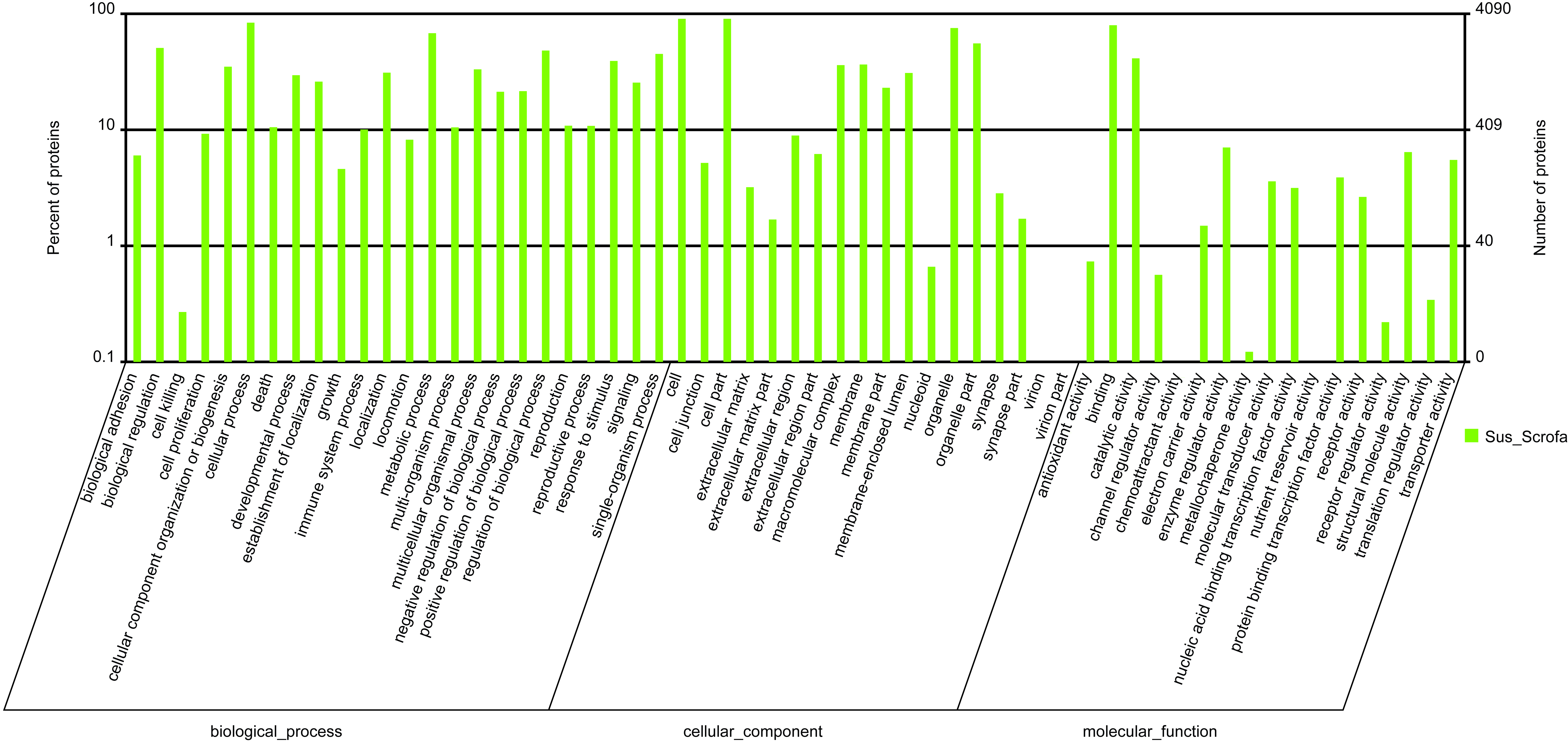

Supplement: Additional file 6: Figure S4. — GO categories of identified proteins. (TIF 1081 kb) [file 12864_2016_2464_MOESM6_ESM.tif]

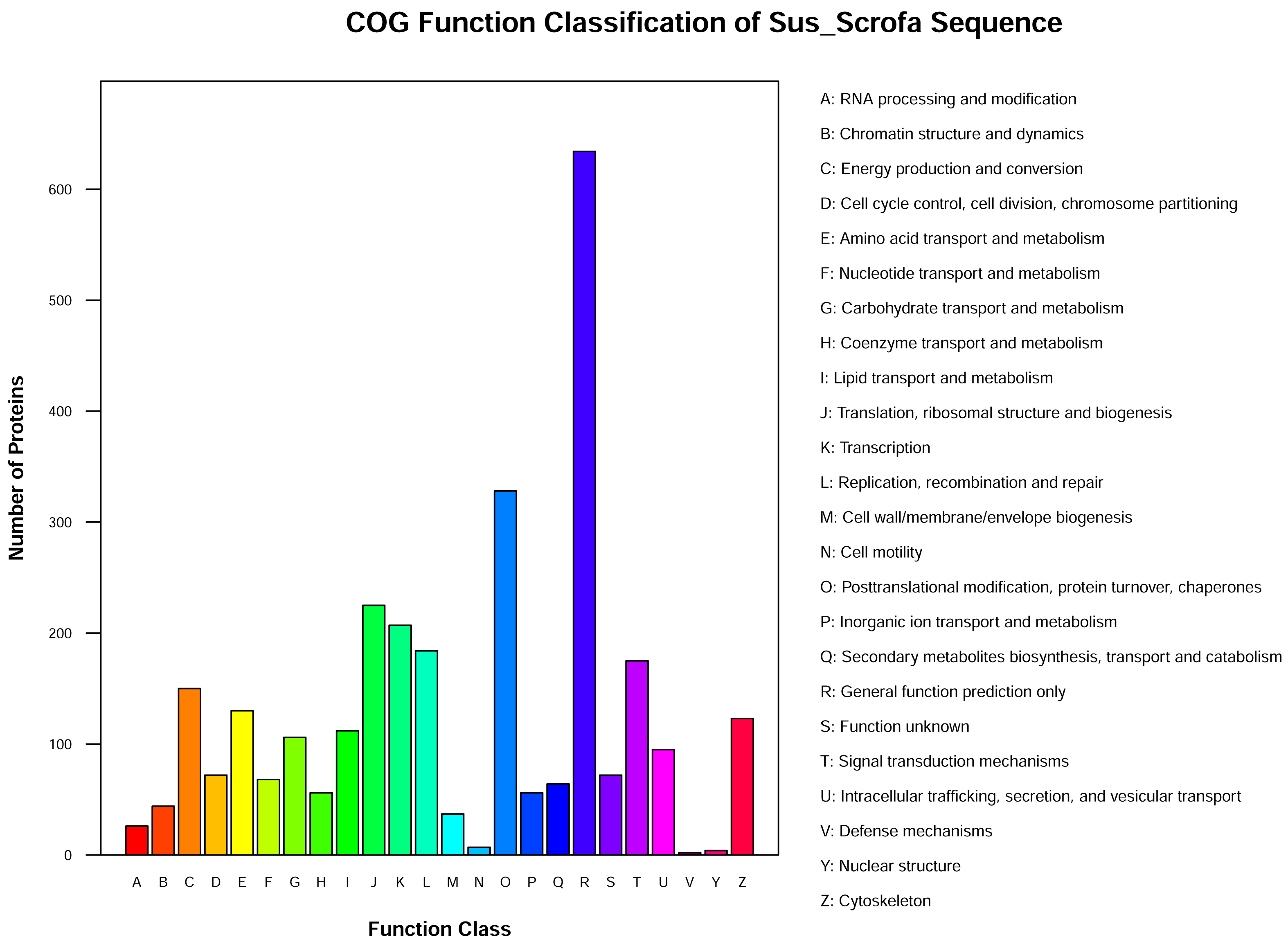

Supplement: Additional file 7: Figure S5. — COG function classification of identified proteins. (TIF 416 kb) [file 12864_2016_2464_MOESM7_ESM.tif]

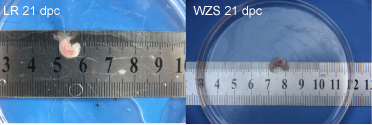

Supplement: Additional file 14: Figure S6. — Embryo picture of 21 dpc (LR&WZS). (TIF 164 kb) [file 12864_2016_2464_MOESM14_ESM.tif]
